# O02 Patterns of GP and nurse-independent prescriber prescriptions for antibiotics dispensed in the community in England: a retrospective analysis

**DOI:** 10.1093/jacamr/dlad066.002

**Published:** 2023-06-14

**Authors:** Molly Courtenay, David Gillespie, Rosemary Lim

**Affiliations:** School of Healthcare Sciences, Cardiff University, Cardiff, UK; Centre for Trials Research, School of Medicine, Cardiff University, Cardiff, UK; Reading School of Pharmacy, University of Reading, Reading, UK

## Abstract

**Background:**

The substitution of nurses for doctors is a strategy used in primary care to improve access to, and efficiency and quality of, care. Many of these nurses prescribe medicines including antibiotics.

**Objectives:**

To identify nurse-independent prescriber (NIP) and GP numbers in England, the proportions and types of NIPs and GP antibiotic prescriptions dispensed in the community, and the impact of COVID-19 on the volume, rate and types of antibiotic prescriptions dispensed.

**Methods:**

A descriptive population-based retrospective cohort study using data obtained from the National Health Service Business Service Authority (under a Freedom of Information request) on GP and nurse-independent prescriber prescriptions for antibiotics dispensed in the community in England between January 2014 and October 2021.

**Results:**

Between 2014 and 2021, the number of NIPs (headcount) in England almost doubled from 24 457 to 41 649. Of the 41 649 NIPs in 2021, the prescriptions of 34 997 (84%) were dispensed in the community, i.e. a rise of 146% over this 6-year period. By contrast, GP numbers (headcount) rose by 10% to 44 681. Of the 25.373 million antibiotic prescriptions dispensed between 2014 and 2021, NIPs were responsible for 8.6%. The rate of dispensed antibiotic prescriptions decreased (NIPs by 50% and GPs by 21%) between 2014 and 2020 (where data across the full year were available). This decreasing trend continued following the onset of the pandemic across both groups. In line with guidance for the treatment of minor infections, narrow-spectrum antibiotics (penicillins, macrolides and tetracyclines) were the most frequently dispensed across both NIPs and GPs. Trends in prescription of antibiotics during the pandemic were reflected in the use of individual classes of antibiotics prescribed for RTIs.

**Conclusions:**

Given the rising numbers of nurse prescribers and their increasing contributory influence on total antibiotic prescribing, it is important that this group is included in antimicrobial stewardship efforts. NIPs prescribe fewer antibiotics than GPs but work under different conditions (e.g. longer consultations and less complex patients), and so, interventions should also be tailored to this group and the population and context in which they are delivered.

